# Bioactive Phenolic Compounds from Lingonberry (*Vaccinium vitis-idaea* L.): Extraction, Chemical Characterization, Fractionation and Cellular Antioxidant Activity

**DOI:** 10.3390/antiox11030467

**Published:** 2022-02-26

**Authors:** Tina Kostka, Johanna Josefine Ostberg-Potthoff, Joachim Stärke, Claudia Guigas, Seiichi Matsugo, Valentin Mirčeski, Leon Stojanov, Sanja Kostadinović Veličkovska, Peter Winterhalter, Tuba Esatbeyoglu

**Affiliations:** 1 Institute of Food Science and Human Nutrition, Gottfried Wilhelm Leibniz University Hannover, Am Kleinen Felde 30, 30167 Hannover, Germany; kostka@lw.uni-hannover.de; 2 Institute of Food Chemistry, Technische Universität Braunschweig, Schleinitzstrasse 20, 38106 Braunschweig, Germany; j.ostberg@tu-braunschweig.de (J.J.O.-P.); p.winterhalter@tu-braunschweig.de (P.W.); 3 Department of Safety and Quality of Fruit and Vegetables, Max Rubner-Institut, Federal Research Institute of Nutrition and Food, Haid-und-Neu-Str. 9, 76131 Karlsruhe, Germany; joachim.staerke@mri.bund.de (J.S.); claudia.guigas@mri.bund.de (C.G.); 4 School of Natural System, College of Science and Engineering, Kanazawa University, Kakuma-Machi, Kanazawa 920-1192, Japan; matsugoh@staff.kanazawa-u.ac.jp; 5 Department of Inorganic and Analytical Chemistry, University of Lodz, Tamka 12, 91-403 Lodz, Poland; valentin.mircheski@chemia.uni.lodz.pl; 6 Institute of Chemistry, Ss. Cyril and Methodius University, Arhimedova 5, 1000 Skopje, North Macedonia; leonstojanov@pmf.ukim.mk; 7 Faculty of Agriculture, University “Goce Delčev”, Krste Misirkov 10-A, 2000 Štip, North Macedonia; sanja.kostadinovik@ugd.edu.mk

**Keywords:** juice, polyphenol, anthocyanin, ESR spectroscopy, cyclic voltammetry, HepG2 cells, LC-MS, TEAC, ROS, DPPH

## Abstract

Lingonberries contain high contents of bioactive compounds such as chlorogenic acids and anthocyanins. In addition to radical scavenging and antioxidant activities, these compounds can protect cells from DNA damage. For this reason, lingonberries might be well suited for nutraceuticals or natural biomedicines. To assess these applications, the present study characterized and identified the most effective extract, only consisting of anthocyanins, copigments or a mixture of both, obtained from a lingonberry juice concentrate. An extract was generated by using a XAD-7 column followed by fractionation into anthocyanins and copigments using adsorptive membrane chromatography. After identification of main polyphenols by HPLC–photodiode array–electrospray ionization–tandem mass spectrometry, free radical scavenging activity was analyzed by electron spin resonance spectroscopy using 2,2-diphenyl-1-picrylhydrazyl and galvinoxyl radicals. Furthermore, cyclic voltammetry analyses and the Trolox equivalent antioxidant capacity (TEAC) assay were applied. Finally, the reactive oxygen species (ROS) reducing effects of the lingonberry extract and its fractions were evaluated in HepG2 cells. While the combination of anthocyanins and copigments possessed the highest antioxidant activities, all samples (XAD-7 extract, anthocyanin and copigment fraction) protected cells from oxidative stress. Thus, synergistic effects between phenolic compounds may be responsible for the high antioxidant potential of lingonberries, enabling their use as nutraceuticals.

## 1. Introduction

Human cells are constantly exposed to compounds and molecules threatening their viability. For instance, reactive oxygen species (ROS) originating from cellular respiration in mitochondria [1] or exogenous factors, such as ultraviolet light originating from sunlight, inducing up to 100,000 lesions per hour in exposed cells [2], contribute significantly to DNA damage in human tissues. Antioxidants are important counterparts in these processes and especially against ROS, by radical scavenging and the prevention of toxic effects induced by oxidative stress [3]. It is well known that fruits and fruit juices are rich in natural antioxidants, e.g., phenolic compounds, which possess several health improving effects, such as radical scavenging activities or the prevention of DNA damage [4,5,6,7,8]. Due to these bioactive phenolic compounds, berries have been recognized as a “superfood” [9], while lingonberries possess one of the highest phenolic contents compared to other berries [10]. Wang and colleagues (2000, 2000, 2003, 2005) analyzed the total phenolic content as well as the antioxidant potential of several berries, including cranberries, blackberries, strawberries, blueberries, raspberries and lingonberries [4,5,11,12]. Compared to other berries, lingonberries possessed the highest antioxidant activity [11,12]. Similar results for the content of phenolic compounds were documented by Vyas et al., (2013) [13], showing higher contents of total phenolics, anthocyanins and flavonols for lingonberries compared to blueberries.

Lingonberry (*Vaccinium vitis-idaea* L.; family: Ericaceae) is widely distributed in Nordic Eurasia and possesses the morphological characteristics of a perennial evergreen dwarf shrub. Especially in Scandinavia, lingonberries are traditionally used as bioactive foods rich in vitamins and antioxidants [14,15]. Lingonberry extracts, obtained by the extraction and concentration of phenolic compounds from fruits, showed high antioxidant activities against ROS (peroxyl, hydroxyl, oxygen radicals) and more specific radicals used in the 2,2-diphenyl-1-picrylhydrazyl (DPPH) and oxygen radical absorbance capacity (ORAC) assay [11,16,17]. Moreover, antidiabetic effects [18,19], antimicrobial effects [20] and even a DNA damage prevention potential [21] have been described for lingonberries. Thus, lingonberries seem to be one of the most effective promising fruits, combining several health improving properties. Nevertheless, these effects depend on individual consumption as well as the uptake of bioactive compounds in the gut. Most phenolic compounds are less soluble in water, while their bioavailability and final functionality are limited, as reviewed by several authors [22,23,24]. For instance, more than several hundred kilograms of red grapes or apples need to be consumed to reach daily effective doses of polyphenols [23]. Therefore, the consumption of fruit extracts in the form of nutraceuticals and not the consumption of whole fruits should be practiced for higher effects. To go one step further, the most effective compounds, or fractions, should be identified and isolated to optimize and promote the development of new biomedicines. Antioxidant potential and the behavior towards free radicals differ between individual phenolic compounds, e.g., the anthocyanin cyanidin-3-*O*-glucoside showed an antioxidant activity twice as high as chlorogenic acid [12,25]. It is hypothesized that molecular structure, more precisely hydroxyl substituents, are responsible for these differences [12], while some synergistic effects have been discussed, likewise [26].

To shed some light on this substance dependent bioactivity, lingonberry phenolics were enriched and fractionated into an anthocyanin and copigment fraction, followed by analyzing the phenolic composition using HPLC-PDA-ESI-MS/MS. The different fractions were compared to each other as well as to the entire extract, consisting of both fractions, regarding their antioxidant potential in chemical assays, cyclic voltammetry and in in vitro studies using HepG2 cells.

## 2. Materials and Methods

### 2.1. Cell Culture

Human hepatoma cells (HepG2 cells; DSMZ-No: ACC-180) were purchased from the German Collection of Microorganisms and Cell Cultures (Braunschweig, Germany). Cell culture medium consisted of Roswell Park Memorial Institute (RPMI) 1640 medium supplemented with L-glutamine, 2.0 g/L NaHCO_3_ (all obtained from PAN Biotech, Aidenbach Germany), 10% (*v*/*v*) fetal bovine serum (Fisher Scientific, Schwerte Germany), 100 IU/mL penicillin and 100 µg/mL streptomycin. The cells were kept at 37 °C with 5% CO_2_ in a humidified atmosphere by changing the medium every 2–3 days.

### 2.2. Lingonberry (Vaccinium vitis-idaea L.) Sample

Lingonberry juice concentrate (JC; *Vaccinium vitis-idaea* L.) was obtained from Symrise AG (Holzminden, Germany).

### 2.3. Preparation of the Lingonberry Extract by Amberlite^®^ XAD-7 and Fractionation Using Adsorptive Membrane Chromatography

According to Kostka et al. (2020), carbohydrates, organic acids and minerals were removed from lingonberry JC, while phenolic compounds were enriched. A detailed scheme and protocol for the XAD-7 fruit extract preparation and fractionation by membrane chromatography is given in Kostka et al. (2020) [27]. Briefly, lingonberry JC (about 900 mL) was applied onto a preconditioned Amberlite^®^ XAD-7 column (100 × 7 cm; Amberlite^®^ XAD-7 was obtained from Sigma-Aldrich, Steinheim, Germany). Undesirable compounds were removed from the column by washing with double deionized water, followed by the elution of the retained phenolic compounds using a 19:1 (*v*/*v*) ethanol/acetic acid solution (both p.a. grade; ethanol was obtained from Sigma-Aldrich; acetic acid was ordered from VWR, Leuven, Germany). Finally, XAD-7 extract was freeze-dried and the solvents evaporated at low pressure below 40 °C. Thus, about 41 g lingonberry XAD-7 extract was obtained.

For fractionation, a XAD-7 solution, prepared using ethanol/acetic acid (19:1; *v*/*v*), was applied onto a cellulose membrane adsorber (type Sartobind S IEX 150 mL) from Sartorius (Göttingen, Germany) and eluted stepwise. Copigments, defined as non-colored phenolic substances, were obtained by using a 19:1 (*v*/*v*) ethanol/acetic acid solution, while for anthocyanins a mixture consisting of ethanol and an aqueous 1 N NaCl solution (1:1; *v*/*v*; NaCl, 99%, p.a., was purchased from Carl Roth, Karlsruhe, Germany) was used. Similar to the XAD-7 extract, the anthocyanin fraction (AF) and copigment fraction (CF) were freeze dried. To prevent interfering effects, NaCl was removed from AF by a further XAD-7 purification [27].

### 2.4. Quantification of the Total Phenolic Content (TPC)

The TPC of lingonberry JC, XAD-7 extract as well as AF and CF was measured by using the Folin–Ciocalteu assay according to Singleton and Rossi (1965) [28] with slight modifications. Briefly, solutions of 1 mg/mL of XAD-7 extract, AF or CF were prepared, while JC was directly used. Water served as blank. One milliliter of Folin–Ciocalteu reagent (diluted 1:10 with water; *v*/*v*; Merck, Darmstadt, Germany) was mixed with 200 µL of sample solution) and incubated at room temperature for 7 min. Afterwards, 800 µL Na_2_CO_3_ solution (7.5%; 99%, p.a., Carl Roth, Karlsruhe, Germany) was added, followed by a further incubation at room temperature for 2 h. Finally, absorption at *λ* = 760 nm was measured using a V-750 UV-visible/NIR spectrophotometer (Jasco, Gross-Umstadt, Germany). For TPC calculation, a standard curve of 11–66 mg/L gallic acid (Fluka, Buchs, Switzerland) was analyzed, while all samples were expressed as grams of gallic acid equivalents (GAE) per 100 g freeze-dried extract.

### 2.5. UHPLC-PDA Analyses for Polyphenols Quantification

The lingonberry XAD-7 extract was analyzed for anthocyanins, flavonols and phenolic acids using ultra high performance liquid chromatography with photodiode array, including a Zorbax Eclipse Plus C18 (1.8 µ, 50 mm × 2.1 mm) column (Agilent, Waldbronn, Germany) with a precolumn in a 1290 Infinity II UHPLC system (Agilent, Waldbronn, Germany). For quantification, the following wavelengths were used: *λ* = 520 nm for anthocyanins (cyanidin-3-*O*-glucoside as standard compound), *λ* = 360 nm for flavonols (quercetin-3-*O*-glucoside as standard compound) and *λ* = 320 nm for phenolic acids (chlorogenic acid as standard compound). Details about the flow rate, gradients and limit of quantification are given in Kostka et al. (2020) [27].

### 2.6. Identification of Polyphenols by HPLC Photodiode Array Electrospray Ionization Tandem Mass Spectrometry (HPLC–PDA–ESI–MS/MS)

The characterization and identification of polyphenols was performed by coupling an HPLC system (1100/1200 series, Agilent, Waldbronn, Germany) to a high capacity spherical trap Ultra Ion Trap mass spectrometer (Bruker Daltonics, Bremen, Germany) using an electrospray ionization source. Detailed information about the method is written in Ostberg-Potthoff et al. (2019) [29].

### 2.7. Detection of Radical Scavenging Activities by the Trolox Equivalent Antioxidant Capacity (TEAC) Assay

The TEAC assay was performed as described by Kostka et al. (2020) [27]. Therefore, sample solutions of 1 mg/mL of the three extracts (XAD-7, AF, CF) were prepared in ethanol, while lingonberry JC was used without any dilutions in the assay. Twenty microliters of sample solution were mixed with 1 mL ABTS working solution (ABTS: 2,2′-Azino-bis-(3-ethylbenzothiazolin-6-sulfonic acid; 98%, Sigma-Aldrich, St. Louis, MO, USA) and incubated for 12 min at room temperature. The ABTS stock solution consists of 7 mM ABTS and 2.5 mM K_2_S_2_O_8_ (Riedel-de-Haën, Seelze, Germany) in water, which was diluted with ethanol to get the ABTS working solution. Thereafter, the stock solution was added dropwise to 25 mL ethanol until the absorption reached a value of 0.7. After incubation, the absorbance at *λ* = 734 nm was measured and calculated with a Trolox standard curve (Trolox: 6-hydroxy-2,5,7,8-tetramethylchromane-2-carboxylic acid; 98% purum, Fluka, Buchs, Switzerland), which was dissolved in ethanol, serving as an equivalent substance for quantification of radical scavenging activities. Thus, the results were expressed as millimoles of Trolox equivalents (TE) per 100 g or 100 mL sample.

### 2.8. Detection of Radical Scavenging Activities by Electron Spin Resonance (ESR) Spectroscopy

ESR analyses were achieved according to Kostka et al. (2020) [27] using a JEOL JES-FR30 EX free radical monitor (Jeol Ltd., Akishima, Japan). Briefly, all samples were tested for scavenging activities against DPPH (final concentration: 1 mM) and galvinoxyl (final concentration: 0.5 mM) radicals (both obtained from Tokyo Chemical Industry, Tokyo, Japan). Therefore, an extract or fraction specific dose response curve from 0–100 µg/mL was monitored regarding the decrease in radical signal intensities. All experiments were performed in triplicate and normalized to untreated control (0 µg/mL). For each sample, IC_50_ with standard deviation was calculated by nonlinear regression analyses using Prism (version 9.2.0; GraphPad, La Jolla, CA, USA) with the following parameters: F = 50, Top = 100, Bottom = 0.

### 2.9. Cyclic Voltammetry for the Evaluation of the Antioxidant Capacity

Cyclic and square wave voltammetry were used for the determination of the antioxidant capacity of the three lingonberry samples. All electrochemical measurements were performed using potentiostat/galvanostat Palm Sens Instrumentation (PalmSens 3, Houten, The Netherlands), controlled by PCT race version 3.0.6 software (Houten, The Netherlands) [30]. Briefly, the antioxidant capacity of the lingonberry XAD-7 extract and its fractions were determined by measuring of 1 mg/mL samples diluted in phosphate buffer using a glassy carbon working electrode (3 mm-ϕ). An Ag/AgCl (3 mol/L KCl) (i.e., silver/silver chloride electrode, with 0.1 M phosphate buffer, pH = 7.3) served as reference electrode, while platinum wire was used as a counter electrode. Before each measurement, the working electrode was cleaned by polishing with aluminum oxide (Al_2_O_3_) powder for proximately 1 min, followed by rinsing with ddH_2_O, acetone and air-drying. The conditions were as follows: E1 = 0 V, v = 10 mV/s and Estep = 1 mV. All experiments were performed at room temperature.

### 2.10. Preparation of Sample Ssolutions for HepG2 Cell Culture Treatment

For in vitro analyses, freeze-dried extracts and fractions were dissolved in dimethyl sulfoxide (DMSO; Merck, Kenilworth, NJ, USA) to stock solutions of 100 mg/mL, respectively. Thus, DMSO is the solvent control in the in vitro assays. Before cell treatment, stock solutions were sterile filtered (0.22 µm) to prevent contaminations.

### 2.11. Detection of Sample-Induced Cytotoxic Effects Using 3-(4,5-Dimethylthiazol-2-yl)-2,5-diphenyltetrazolium bromide (MTT) Viability Assay

Cytotoxic effects induced by lingonberry extracts were excluded by monitoring cell viability in the MTT cell viability using the MTT Cell Proliferation Kit (Roche, Mannheim, Germany) according to Kostka et al. (2020) [27]. HepG2 cells were treated with concentrations of 75 and 100 mg/mL for 24 h or as positive control with 1% Triton X (Serva Electrophoresis, Heidelberg, Germany), resulting in high rates of cell death. The MTT assay protocol was performed as recommended by manufacturer instructions. The reduction in the MTT reagent, which correlates with cell metabolism, was measured at *λ* = 595 nm and calculated as relative cell viability compared to solvent control.

### 2.12. Labeling of Reactive Oxygen Species (ROS) Positive Cells for Detection of Antioxidant Effects

The lingonberry extract and its fractions were analyzed for antioxidant effects in a two step treatment of HepG2 cells, as described by Kostka et al. (2020) [27]. Briefly, HepG2 cells (1 × 10^6^ cells per well in 6-well plates (Greiner Bio-one, Frickenhausen, Germany)) were seeded and incubated for 24 h. Afterwards, the cells were treated for 1 h with 100 µg/mL lingonberry XAD-7 extract, its fraction or 0.1% DMSO as solvent control, diluted in Hanks’ balanced salt solution (HBSS; PAN Biotech, Aidenbach, Germany). Afterwards, the cells were incubated with a nontoxic concentration of H_2_O_2_ (100 µM) for 1 h to induce oxidative stress. To exclude lingonberry induced oxidative stress, HepG2 cells were treated for 2 h with lingonberry samples without using H_2_O_2_. All treated cells were washed, trypsinized, centrifuged and resuspended in 200 µL of the assay buffer Muse Oxidative Stress Kit (Merck, Kenilworth, NJ, USA). Then, 10 µL of the cell suspension were diluted with 190 µL working solution of the Kit, followed by incubation at 37 °C for 30 min. Finally, the suspension was analyzed by the Muse Cell Analyzer (Merck, Kenilworth, NJ, USA), which automatically counted and calculated the content of ROS positive and ROS negative cells.

### 2.13. Statistics

Statistical analysis was performed using Prism (version 9.2.0; GraphPad, La Jolla, CA, USA). Data were analyzed by Shapiro–Wilk test for normal distribution, followed by a one way ANOVA with Tukey’s multiple comparison test (*p* < 0.05). Results are shown as mean ± standard deviation (SD) of at least three independent experiments.

## 3. Results

### 3.1. Characterization of Lingonberry Juice, XAD-7 Extract, AF and CF

**Figure 1:**
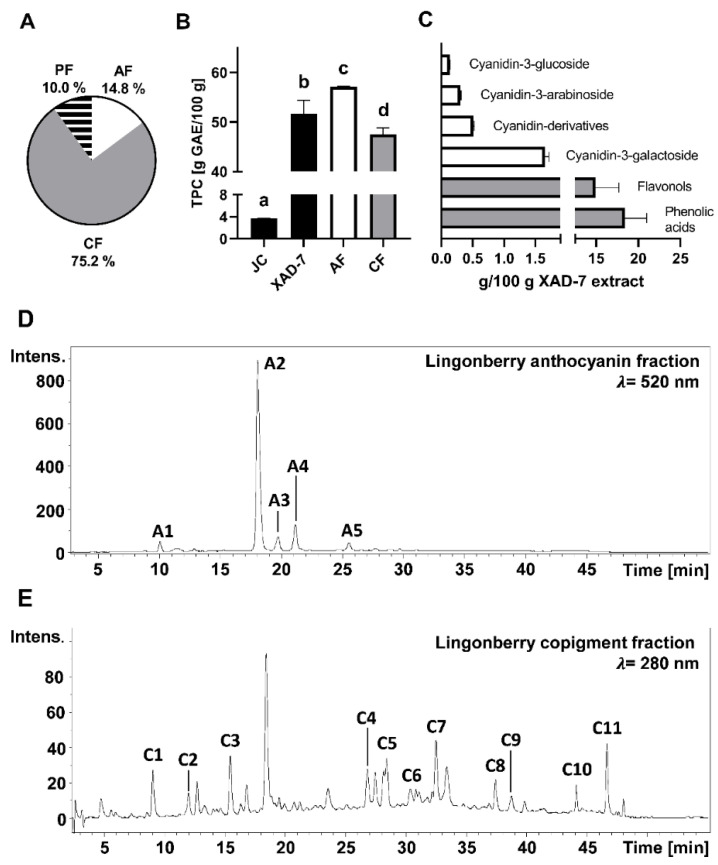
Chemical characterization of lingonberry juice concentrate (JC), the XAD-7 extract, the anthocyanin fraction (AF) and the copigment fraction (CF). Mean ± standard deviation; n = 3. (**A**) Composition of lingonberry XAD-7 extract. (**B**) Total phenolic content (TPC) of JC, XAD-7 extract, AF and CF. Different letters represent statistically significant differences (*p* < 0.05) within the samples. (**C**) Concentration of the main phenolic compounds in the XAD-7 extract. (**D**) HPLC chromatogram of AF. (**E**) HPLC chromatogram of CF. Identification of HPLC peaks is given in Table 1. Intens.: signal intensity (mAU).

In total, 41.0 g of a XAD-7 extract were obtained from 1.2 kg lingonberries *(Vaccinium vitis-idaea* L.) juice concentrate (JC), which was further separated into anthocyanins and copigments by adsorptive membrane chromatography. The XAD-7 extract consisted of 14.8% anthocyanin fraction (AF) and 75.2% copigment fraction (CF). About 10.0% of the XAD-7 extract applied to the membrane chromatography was retained on the membrane, which could be present the polymeric compounds (Figure 1A). While lingonberry JC contained an average TPC of 3.70 g GAE/100 g, the TPC of XAD-7 extract as well as of AF and CF were significantly higher (*p* < 0.0001) and varied from 47.5–57.1 g GAE/100 g (Figure 1B). Moreover, AF showed the highest TPC, which was significantly higher than XAD-7 (*p* < 0.05) and CF (*p* < 0.001). TPC in CF was significantly lower (*p* < 0.05) compared to the XAD-7 extract. Phenolic compounds in the XAD-7 extract were characterized by HPLC-ESI-MS/MS analyses, which revealed phenolic acids such as chlorogenic acid (*m*/*z* 353) and flavonols as the most abundant phenolic compounds (Figure 1C). The concentration of identified anthocyanins in the XAD-7 extract was 2.44 g/100 g and comprised two unknown cyanidin derivatives (*m*/*z* 477 and 737) as well as cyanidin-3-*O*-galactoside (*m*/*z* 449), cyanidin-3-*O*-glucoside (*m*/*z* 449) and cyanidin-3-*O-*arabinoside (*m*/*z* 419). Corresponding HPLC chromatograms for AF and CF are shown in Figure 1D,E. Peak identification is given in Table 1. The data show that the phenolic compounds from lingonberry JC were concentrated in the XAD-7 extract as well in both of its subfractions, CF and AF, whereby the latter exhibited the highest TPC.

### 3.2. Free Radical Scavenging Activity Determined by the TEAC and ESR Assay

**Figure 2:**
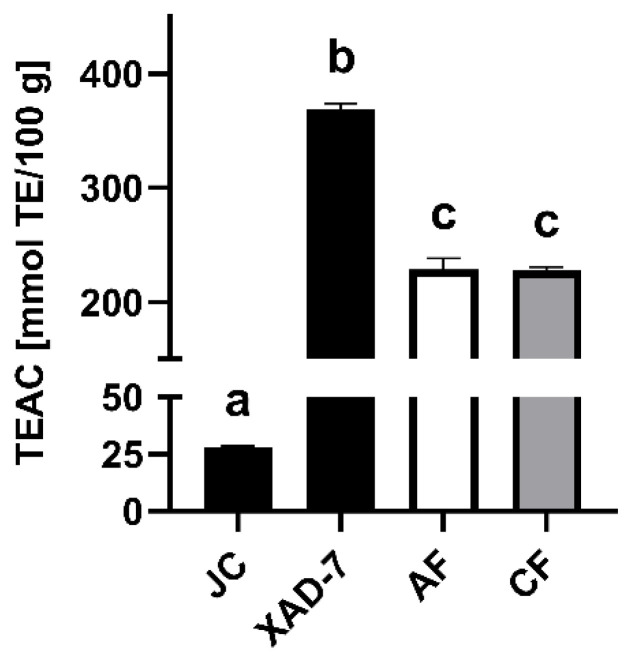
Trolox equivalent antioxidant capacity (TEAC) calculated as Trolox equivalent (TE) per 100 g or 100 mL of lingonberry juice concentrate (JC), XAD-7 extract, its anthocyanin (AF) and copigment fraction (CF). Results are presented as mean ± SD of three independent experiments. Different letters represent statistically significant differences (*p* < 0.0001) within the samples.

The lingonberry extract and its fractions, as well as the JC, were analyzed for their scavenging activity of ABTS^+∙^-radicals in the TEAC assay. While the radical scavenging activity of lingonberry JC was significantly lower (*p* < 0.0001) than that of the generated extracts, AF and CF showed similar results, with a significantly lower capacity (*p* < 0.0001) compared to the XAD-7 extract (Figure 2). The radical scavenging activity of the XAD-7 extract was 1.6-fold higher than its fractions and 13-fold higher than JC.

**Figure 3:**
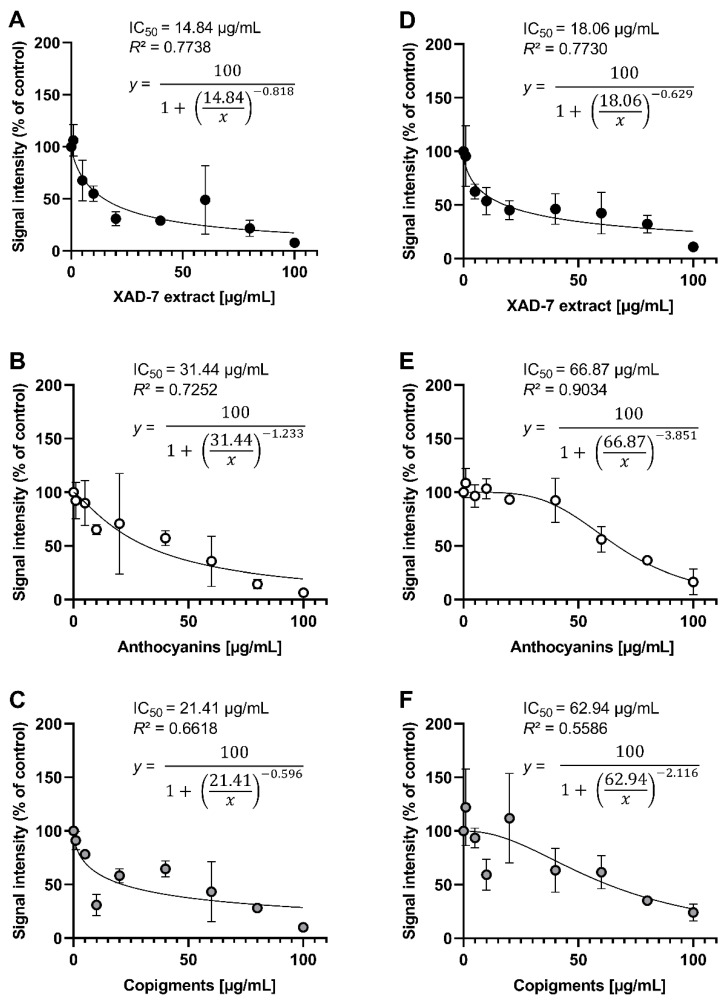
Galvinoxyl radical scavenging activity (**A**–**C**) and DPPH radical scavenging activity (**D**–**F**) of lingonberry XAD-7 extract and its anthocyanin (AF) and copigment fraction (CF). Shown is the mean ± standard deviation, the nonlinear equation curve, as well as the calculated *R*^2^ and *IC*_50_ values of three independent experiments.

The antioxidant potential was additionally examined against galvinoxyl and DPPH radicals by ESR spectroscopy, which revealed comparable results (Figure 3). For galvinoxyl radicals, XAD-7 extract (*IC*_50_ = 14.84 µg/mL; Figure 3A) showed the highest scavenging activity, while higher concentrations of AF (*IC*_50_ = 31.44 µg/mL; Figure 3B) and CF (*IC*_50_ = 21.41 µg/mL; Figure 3C) were needed for reaching 50% signal inhibition. Such differences in the lingonberry extract and its fractions were even higher in DPPH scavenging studies (Figure 3D–F). The inhibition concentrations of AF (*IC*_50_ = 66.87 µg/mL) and CF (*IC*_50_ = 62.94 µg/mL) were 3.5-fold higher than *IC*_50_ of XAD-7 extract (*IC*_50_ = 18.06 µg/mL). In summary, all samples showed dose-dependent radical scavenging activities, whereas the XAD-7 extract exhibited the highest potential in ESR spectroscopy analyses as well as in the TEAC assay.

### 3.3. Electron Transfer Reactions Analyzed by Cyclic Voltammetry

**Figure 4:**
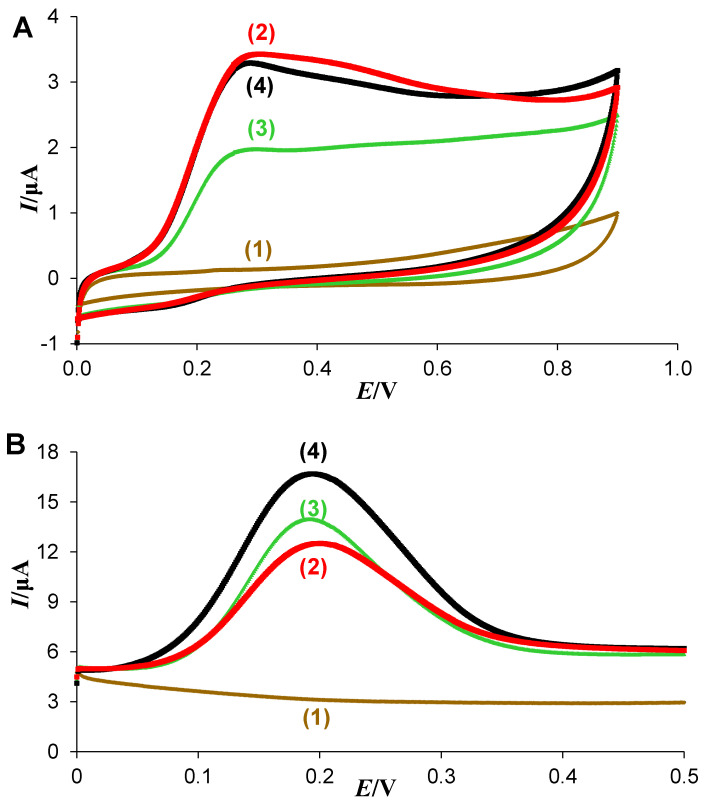
Determination of antioxidant capacity by cyclic voltammetry of lingonberry samples. (**А**) Cyclic voltammograms of lingonberry extracts at concentration of 1 mg/mL in 0.1 M phosphate buffer (pH = 7.3) recorded at potential scan rate of 10 mV/s at glassy carbon electrode. (**B**) Square wave voltammograms. For both panels: blank (1); copigment fraction (2); anthocyanin fraction (3); and XAD-7 (4). The parameters of the potential modulation for square wave voltammograms are: starting potential E_1_ = 0.0 V, square wave frequency *f* = 10 Hz, the height of the potential pulses *E*_sw_ = 50 mV and the step potential ∆*E* = 1 mV.

The lingonberry XAD-7 extract and its fractions (CF and AF) were analyzed for antioxidant capacity by cyclic voltammetry independent of the addition of radicals such as ABTS or DPPH. The results from the cyclic and square wave voltammetry are presented in Figure 4. Compared to the solvent control, lingonberry samples showed a current increase and had the ability to exchange electrons with the electrode. While the shape from cyclic and square wave voltammetry appeared similar for XAD-7 extract and its fractions, lingonberry samples differ in current intensity. In cyclic voltammograms, CF and XAD-7 extract showed the highest intensities. The results of AF were lower, nevertheless, antioxidant activity was detected. In square wave voltammograms, the ability to transfer electrons was as follows: XAD-7 extract > AF > CF. Phosphate buffer as solvent control had no effects. To sum up, lingonberry extracts showed electron transfer reactions with the highest potential for the XAD-7 extract.

### 3.4. Cellular Antioxidant Potential of the Lingonberry Extract and Its Fractions by Reduction of ROS

**Figure 5:**
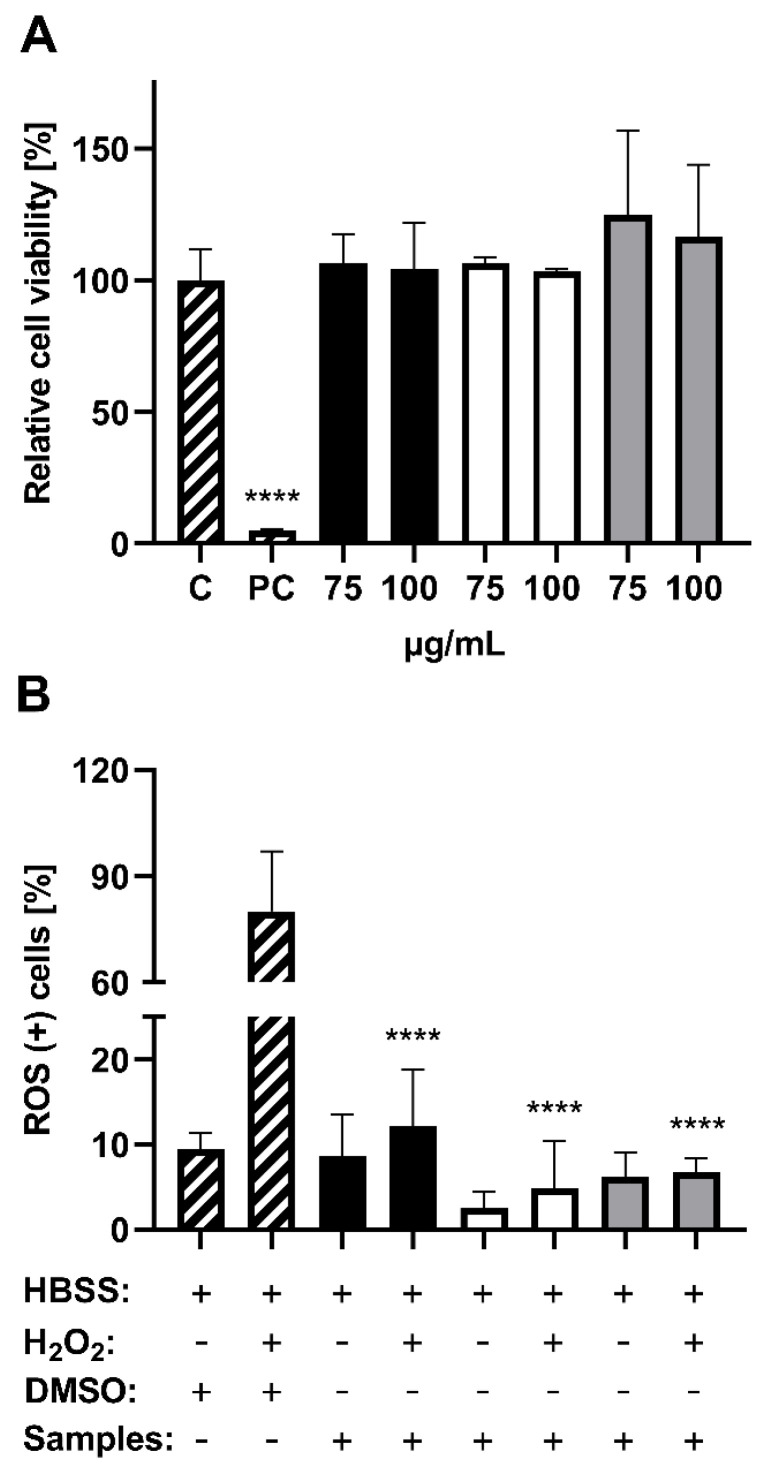
Exclusion of cytotoxic effects as well as the detection of a reactive oxygen species (ROS) reducing potential of lingonberry XAD-7 extract (full black color), anthocyanin fraction (full white color), and copigment fraction (full grey color) using HepG2 cells. Shown is the mean ± standard deviation; n = 3. (**A**) MTT assay; C: 0.1% DMSO as solvent control; PC: 1% Triton X as positive control. Statistically significant differences (**** *p* < 0.0001) compared to solvent control cells. (**B**) ROS reducing effects of lingonberry samples (100 µg/mL) or 0.1% DMSO followed by incubation with or without H_2_O_2_ (100 µM) all dissolved in HBSS. Significance detection: H_2_O_2_ treated or nontreated sample free control (striped color) were compared to samples with or without H_2_O_2_ post-treatment. **** *p* < 0.0001.

To reveal radical scavenging activities in in vitro studies, HepG2 cells were treated with the lingonberry extract and its fractions, prior to ROS induction using H_2_O_2_. Previously, substance induced cytotoxic effects were excluded by dose-finding studies. Concentrations of up to 100 µg/mL of XAD-7 extract and its fractions showed no cytotoxicity in HepG2 cells (Figure 5A). Moreover, CF induced a slightly higher, but nonsignificant increase in cell viability. In consideration of the results of radical scavenging activity assays as well as the cytotoxicity test, all lingonberry samples were tested at concentrations of 100 µg/mL in HepG2 cells. The sole incubation of cells with lingonberry extract and fractions revealed no ROS-inducing potential by the compounds themselves, whereas AF even tended to reduce (non-significantly) the percentage of ROS positive cells (Figure 5B). While H_2_O_2_ led to significantly higher levels of ROS positive cells, with an average of 80%, these effects were significantly reduced (*p* < 0.0001) by all samples, to 4.12%. Thus, XAD-7 extract and its fractions did not differ from each other and were comparable with untreated control cells. To sum up, treatment of HepG2 cells with all the lingonberry samples used in this study resulted in a statistically significant reduction in H_2_O_2_ induced ROS generation, whereby no fraction dependent effects were detected.

## 4. Discussion

In contrast to previously published results, which mainly focused on the phenolic compounds of lingonberry leaves and fruits [13,31,32,33,34,35,36,37], in the present study a commercially available lingonberry juice concentrate was analyzed. To the best of our knowledge, this is the first study directly comparing the antioxidant potential of lingonberry JC with its extract and the separated fractions to identify the most effective compounds and/or possible synergistic effects. Moreover, for the first time, lingonberries (*Vaccinium vitis-idaea* L.) were investigated using cyclic voltammetry as well as ESR analyses.

In lingonberry JC, CF is the most abundant fraction followed by AF and a polymeric fraction (PF), which is comparable to other red fruits. For instance, in red grape JC the copigments were the most abundant fraction (49%), while AF and PF were similar, with a content of 24% AF and 27% PF [29]. Compared to pomegranate juice, which is known as a “superfood” and consisted of 10.8% AF, the content of anthocyanins in lingonberry JC is higher, with a lower content of polymeric compounds (10% in lingonberry JC, 14.3% in pomegranate juice) [27]. Similar to the higher AF content in lingonberry JC, the TPC content is even higher than in pomegranate juices. TPC values from fruit juices such as black cherry, cranberry and blueberry juice are in the range of 50–400 mg GAE/100 mL [38], while the used lingonberry JC possessed a 10-fold higher content of 3700 mg GAE/100 mL. Even in contrast to other studied lingonberry juices, e.g., 95–985 mg GAE/100 mL [11] or 270 mg GAE/100 mL [20], the JC of the present study was rich in phenolic compounds.

First, by using a JC instead of juice, higher values of TPC were expected. Normally, juice concentrates contain a 6-fold higher concentration compared to fruit juices. Therefore, the TPC of the JC was much higher than the TPC of the analyzed juices in the literature. Nevertheless, by considering this dilution factor, the JC of the present study would show a TPC of 617 mg GAE/100 mL lingonberry juice, which is comparable with the literature. The high deviation of TPC in lingonberry juices may have several reasons. On the one hand, the variation in TPC between lingonberry juices correlates with the fruit content, while, on the other hand, the TPC depends on seasonal and cultivar specific differences [35,36,37]. For instance, the TPC of lingonberry fruits ranged from 364–660 mg GAE/100 g [12,35,36]. The aim of the present study was to verify the concentration and enrichment of phenolic compounds, which results in a significantly 10-fold higher TPC for the XAD-7 extract and all fractions compared to lingonberry JC. Similar to the JC, the TPC of the extract and its fractions, with an average of 50 g GAE/100 g, is higher than the extracts found in the literature (36–41 mg GAE/100 g; [39]). These results highlight the efficiency of the used extraction and isolation methods of the present study, as well as the application of juice concentrates for nutraceuticals. Moreover, the fractionation of AF and CF seems reasonable, due to the significantly higher TPC in AF and the significantly lower content in CF. Thus, similar differences in their antioxidant potential could be expected.

The main phenolic compounds were identified and quantified by HPLC-PDA and HPLC–PDA–ESI–MS/MS, showing similar results as those described earlier [15,31,34,40,41]. The anthocyanin content of the XAD-7 extract was significantly higher than in lingonberry juice and fruits (2.44 g/100 g vs. up to 65 mg/100 g and 53 mg/100 g; [11,35]). Consequently, the phenolic compounds including anthocyanins were concentrated in the XAD-7 extract. As discussed earlier [10,12,15,37,42], cyanidin-3-*O*-galactoside (68%) was the most abundant compound in lingonberries, followed by cyanidin-3-*O*-arabinoside (12%) and cyanidin-3-*O*-glucoside (6%). The composition of anthocyanins agreed with the literature, which documented a relative occurrence of 79–92% for cyanidin-3-*O*-galactoside, 11–13% for cyanidin-3-*O*-arabinoside and 5–10% for cyanidin-3-*O*-glucoside [34,35,40,41,43]. Another important group are the flavonols, such as quercetin-3-*O*-galactoside [44]. While lingonberry fruits consist of 163 mg flavonols per 100 g [45], in the recent study the content was concentrated up to 15 g/100 g in the XAD-7 extract. This is in contrast to the results of Mane et al. (2011), who analyzed a commercially available lingonberry extract that consisted of 3 g flavonols per 100 g extract, and, therefore, is less efficient than the method of the present study [15]. To sum up, the extraction of phenolic compounds from lingonberry JC using a XAD-7 column is one of the most efficient methods for the enrichment of phenolic compounds.

The radical scavenging activities by the TEAC assay as well as the cyclic voltammetry of the lingonberry extract and its fractions were analyzed. The shape of the signal from cyclic and square wave voltammetry indicated the oxidation of similar chemical compounds in all extracts examined. More precisely, the shape of the signal from voltammograms implies the oxidation of compounds that include *O*-dihydrobenzene (catechol) in their structure to *O*-quinone and then reversed reduction in the catechol form [46]. The intensity of the signal could be an indicator of the antioxidant potential of the extracts. The results from our study showed that lingonberry extracts obtained by XAD-7 and CF had the highest antioxidant potential, while AF had a lower antioxidant potential determined by cyclic voltammetry. The potential of radical scavenging of all samples was also seen in the TEAC assay. The activity of the used lingonberry JC (25 mmol TE/100 mL) was significantly higher compared to other fruit juices, as well as red wine and tea, ranging from 0.36–4.16 mmol TE/100 mL [38]. Moreover, this ability of radical scavenging could be significantly increased by the generation of lingonberry extracts, showing a 10-fold higher antioxidant potential for AF and CF. The results of the XAD-7 extract were even higher (369 mmol TE/100 g) in the TEAC assay. Thus, the use of lingonberry JC, instead of fruits and juice for the extraction of phenolic compounds, results in a significantly higher yield of TPC and higher antioxidant potential in the TEAC assay [16]. Lingonberries are known for their high radical scavenging activity to DPPH and oxygen radicals [11]. Therefore, lingonberry samples were additionally analyzed against DPPH and galvinoxyl radicals. Interestingly, the results of the TEAC assay agreed with the ESR analyses. In both experiments, XAD-7 showed the highest activity, while the antioxidant potential of AF and CF was comparable, with a significantly lower radical scavenging activity than the XAD-7 extract. This low activity may also be responsible for the different curve fitting of AF and CF in the DPPH assay. Conclusively, the extract contained antioxidant compounds that were removed from AF and CF by fractionation and/or some synergistic effects between both of these fractions, leading to the higher antioxidant potential in the XAD-7 extract. Another important point is the TPC of all extracts. XAD-7 showed a moderate TPC, while AF was significantly higher and CF significantly lower. Thus, the phenolic compounds could not be exclusively responsible for the antioxidant and radical scavenging activity of lingonberry extracts. Otherwise, a direct correlation between TPC and TEAC is less likely, because of substance specific antioxidant activities, which depend on the number of hydroxyl groups [12,25,37]. Zheng and Wang (2003) [12] showed that hydroxyl and methoxy group substitutions of anthocyanins influence the substance specific antioxidant activity. Therefore, differences in TPC and antioxidant potential are likely to be based on the structure and content of extract specific compounds.

Finally, the antioxidant activities of the extract and its fractions were confirmed by in vitro assays using HepG2 cells. The lingonberry samples led to a nonsignificant increase in cell viability. Similar results were documented by Kowalska et al. (2019) [41] and Pacheso et al. (2018) [47] for lingonberry extracts, while Migliorini et al. (2019) [48] showed an increase in HepG2 cell proliferation by anthocyanin rich extracts isolated from red chicory. It is hypothesized that phenolic compounds may increase the activity of mitochondrial dehydrogenase, which is responsible for the MTT reduction and discoloration in the assay [49]. After viability testing, all samples were tested as antioxidants preventing the cells from H_2_O_2_ damage. While H_2_O_2_ significantly increased the number of ROS positive cells up to 75%, XAD-7 extract, as well as its fractions, showed high prevention against oxidative stress (Figure 5). The effects of H_2_O_2_ were completely prevented by reaching levels lower or similar to untreated control (10% ROS positive cells). In contrast to radical scavenging activity results, which showed higher effects only for XAD-7 extract, the antioxidant potential in HepG2 cells was comparable for all samples. It has already been shown that anthocyanins, especially the most common anthocyanins in lingonberries, such as cyanidin-3-*O*-glucoside, cyanidin-3-*O*-arabinoside and cyanidin-3-*O*-galactoside, possessed a high preventive potential against oxidative stress and H_2_O_2_ induced apoptosis [42]. Moreover, anthocyanins were able to reduce UV induced DNA damage by ROS scavenging activity [50,51]. Therefore, higher effects of AF than CF were expected in cell culture experiments. Possibly, extract specific differences would have been seen at lower concentrations. Lehtonen et al. (2010) [41], for instance, depicted that concentrations of a 1–5 mg/mL lingonberry extract decreased intracellular ROS in adipocytes dose dependently, while Migliorini et al. (2019) [48] used various colon carcinoma cell lines and documented high antioxidant effects for 10–100 µg/mL of an anthocyanin rich red chicory extract. Thus, it is hypothesized that even lower concentrations of the used lingonberry extracts were sufficient to induce antioxidant effects, whereas the used extraction method is highly efficient and suitable for application in the generation of nutraceuticals.

## 5. Conclusions

To sum up, for the first time, a highly efficient lingonberry extract and its separated fractions composed of subfractions AF and CF were generated and compared to each other. To the best of our knowledge, one of the highest TPC of lingonberry extract compared to the literature was achieved. Although the extract and its fractions possessed a high antioxidant potential, the highest radical scavenging activities in the TEAC assay, cyclic voltammetry as well as ESR spectroscopy (analyzing the inhibition of DPPH and galvinoxyl radicals) were seen with the XAD-7 extract. In future studies, the hypothesized synergistic effects of the different fractions should be verified by several mixed extracts, including the isolation and characterization of polymeric compounds. Moreover, the substance or fraction specific intracellular effects, e.g., the expression of glutathione reductase, glutathione peroxidase and members of the mitogen activated protein kinase (MAPK) pathways, should be analyzed [11,41]. Such results would highlight the most efficient fractions and/or substances as suitable antioxidants and would promote the application of lingonberry extracts as nutraceuticals.

## Figures and Tables

**Table 1:** Compound identification of the anthocyanin (A1–A5) and copigment fraction (C1–C11) of the lingonberry (*Vaccinium vitis-idaea* L.) XAD-7 extract by HPLC-ESI-MS/MS.

Peak	[M+H]^±^	Fragments (*m*/*z*)	Compound
Positive mode			
A1	737	575, 287	unknown cyanidin derivative
A2	449	287	cyanidin-3-*O*-galactoside
A3	449	287	cyanidin-3-*O*-glucoside
A4	419	287	cyanidin-3-*O*-arabinoside
A5	477	287	unknown cyanidin derivative
Negative mode			
C1	153	109	protocatechuic acid
C2	341	179	caffeic acid hexoside
C3	353	191	chlorogenic acid
C4	193	-	ferulic acid
C5	367	205, 161	unknown phenolic acid
C6	355	194	ferulic acid hexoside
C7	463	301	quercetin-3-*O*-galactoside
C8	447	301	quercetin-desoxyhexoside
C9	433	301	quercetin-pentoside
C10	447	301	quercetin-rhamnoside
C11	301	-	quercetin

## Data Availability

The data underlying this article will be shared on reasonable request to the corresponding author as some part of the data is part of a PhD thesis. All other data is contained within the article.
